# The genetic structure of *Aedes aegypti* populations is driven by boat traffic in the Peruvian Amazon

**DOI:** 10.1371/journal.pntd.0007552

**Published:** 2019-09-18

**Authors:** Sarah Anne J. Guagliardo, Yoosook Lee, Amanda A. Pierce, Jacklyn Wong, Yui Yin Chu, Amy C. Morrison, Helvio Astete, Berry Brosi, Gonzalo Vazquez-Prokopec, Thomas W. Scott, Uriel Kitron, Steven T. Stoddard

**Affiliations:** 1 Department of Environmental Sciences, Emory University, Atlanta, Georgia, United States of America; 2 Department of Pathology, Microbiology and Immunology School of Veterinary Medicine, University of California, Davis, California, United States of America; 3 Department of Biology, Emory University, Atlanta, Georgia, United States of America; 4 Department of Entomology and Nematology, University of California, Davis, California, United States of America; 5 Department of Virology and Emerging Infections, Naval Medical Research Unit No.6 (NAMRU-6) Iquitos Laboratory, Iquitos, Loreto, Peru; North Carolina State University, UNITED STATES

## Abstract

In the Americas, as in much of the rest of the world, the dengue virus vector *Aedes aegypti* is found in close association with human habitations, often leading to high population densities of mosquitoes in urban settings. In the Peruvian Amazon, this vector has been expanding to rural communities over the last 10–15 years, but to date, the population genetic structure of *Ae*. *aegypti* in this region has not been characterized. To investigate the relationship between *Ae*. *aegypti* gene flow and human transportation networks, we characterized mosquito population structure using a panel of 8 microsatellite markers and linked results to various potential mechanisms for long-distance dispersal. Adult and immature *Ae*. *aegypti* (>20 individuals per site) were collected from Iquitos city and from six neighboring riverine communities, i.e., Nauta, Indiana, Mazan, Barrio Florida, Tamshiaco, and Aucayo. F_ST_ statistics indicate significant, but low to moderate differentiation for the majority of study site pairs. Population structure of *Ae*. *aegypti* is not correlated with the geographic distance between towns, suggesting that human transportation networks provide a reasonable explanation for the high levels of population mixing. Our results indicate that *Ae*. *aegypti* gene flow among sub-populations is greatest between locations with heavy boat traffic, such as Iquitos-Tamshiaco and Iquitos-Indiana-Mazan, and lowest between locations with little or no boat/road traffic between them such as Barrio Florida-Iquitos. Bayesian clustering analysis showed ancestral admixture among three genetic clusters; no single cluster was exclusive to any site. Our results are consistent with the hypothesis that human transportation networks, particularly riverways, are responsible for the geographic spread of *Ae*. *aegypti* in the Peruvian Amazon. Our findings are applicable to other regions of the world characterized by networks of urban islands connected by fluvial transport routes.

## Author summary

*Aedes aegypti*, the primary mosquito vector of dengue, is a highly invasive species that is expanding from urban to peri-urban and rural areas throughout the Americas. Previous studies documented the role of human transportation networks in *Ae*. *aegypti* long-distance dispersal. We examined whether patterns of *Ae*. *aegypti* gene flow are consistent with this observation. Mosquitoes were collected from seven locations, including the large Amazonian city of Iquitos, Peru, and six neighboring rural communities, and their genetic relatedness was compared using 8 microsatellite markers. Our results showed ample gene flow among mosquito populations in this region, with greater gene flow observed among sites that are connected by fluvial routes. These findings are consistent with the hypothesis that human transportation networks, especially via boats, are a primary contributing factor to the spread of *Ae*. *aegypti* in the Peruvian Amazon.

## Introduction

Anthropogenic activities such as trade and transportation contribute to the unintentional spread of invasive organisms across the globe [1, 2], resulting in serious consequences for public health, agriculture, the economy, and native ecosystems [3–5]. Pathogens and their insect vectors are important examples of invasive organisms that directly impact human health.

The invasive mosquito, *Aedes aegypti*, is the primary vector of dengue, urban yellow fever, and Zika viruses, is an important vector of chikungunya virus [6–8], and is a competent or suspected vector of Mayaro virus [9]. Thought to be African in origin, *Ae*. *aegypti* most likely was transported to the Americas via ships used for the transport of slaves and goods in the 15^th^-19^th^ centuries [10–12]. *Ae*. *aegypti* was first reported in Peru in 1852, but was declared eradicated in 1958 following the success of a large-scale Pan American Health Organization yellow fever control program [13]. The Amazonian city of Iquitos was the first documented site of *Ae*. *aegypti* (in 1984) and dengue (in 1990) reestablishment in Peru [14, 15]. In recent years, *Ae*. *aegypti* mosquitoes have been expanding geographically from urban to peri-urban and rural areas throughout Peru and the rest of Latin America [16–18].

*Ae*. *aegypti* dispersal can occur in one of two ways: the slower, active dispersal of flying adult females in search of bloodmeals or oviposition sites, or the faster, passive human-mediated dispersal, by which humans unintentionally transport mosquitoes via vehicle traffic (boats, cars, planes, etc). The latter can involve transport of eggs, larvae, pupae, or adults, all of which have been documented in vehicles, particularly in boats [10, 19–22]. Our previous research in the Peruvian Amazon demonstrated *Ae*. *aegypti* infestation on different vehicles commonly used for trade and transportation, including large barges, medium-sized barges, and buses [23, 24]. Characterizing the relative role and importance of both active and passive dispersal mechanisms is paramount for understanding vector population structure and the dynamics of pathogen transmission.

Previous studies have characterized *Ae*. *aegypti* population genetic structure at global scales [25–29], in regions within countries [30–32], and within cities [33, 34]. *Ae*. *aegypti* genetic differentiation across various spatial scales is likely due to its limited flight range (rarely exceeding 100m under natural conditions [35–37]), heterogeneities in insecticide application, human population densities, and water storage habits [33, 38, 39]. At coarser scales (i.e., between nations, or regions within nations), human transportation networks have been implicated as a driver of *Ae*. *aegypti* gene flow; that is, relatedness between populations has been shown to correlate with major highways and waterways [25, 32]. The Amazonian city of Iquitos, Peru presents is a unique setting in which to address questions of *Ae*. *aegypti* gene flow and human transit, as people in this region rely heavily on rivers as a means of transport between locations. Iquitos is the site of many longitudinal studies related to dengue epidemiology and *Ae*. *aegypti* ecology [40], yet little is known about its population structure in this region. In this study, we used *Ae*. *aegypti* samples collected from several sites within and around Iquitos to characterize the population structure, and preliminarily explored whether genetic relatedness among mosquito populations is driven by human transportation networks.

## Methods

### Ethics statement

Permission for this study was granted by the Loreto Regional Health Department, and the study protocol was approved by the NAMRU-6 Institutional Review Board in compliance with all applicable federal regulations governing the protection of human subjects (protocol number NAMRU6.2012.0039). In addition, the Emory University Institutional Review Board determined that this study does not represent human subjects based research.

### Study area

Accessible only by plane or boat, Iquitos is surrounded by other, smaller settlements that are primarily connected to one another via river networks. The only significant source of terrestrial transit is the 95km Iquitos-Nauta Highway, connecting Iquitos (population, pop henceforth: 406,340) to the smaller city of Nauta (pop: 13,983) [41]. Once the epicenter of the rubber industry in the early 1900s, the Iquitos economy now relies predominantly on oil and timber exportations in addition to tourism. This setting is ideal for studying the invasion dynamics of *Ae*. *aegypti*, because the region’s inhabitants are dependent on both fluvial and terrestrial routes for trade and transportation.

### Mosquito collections

**Fig 1:**
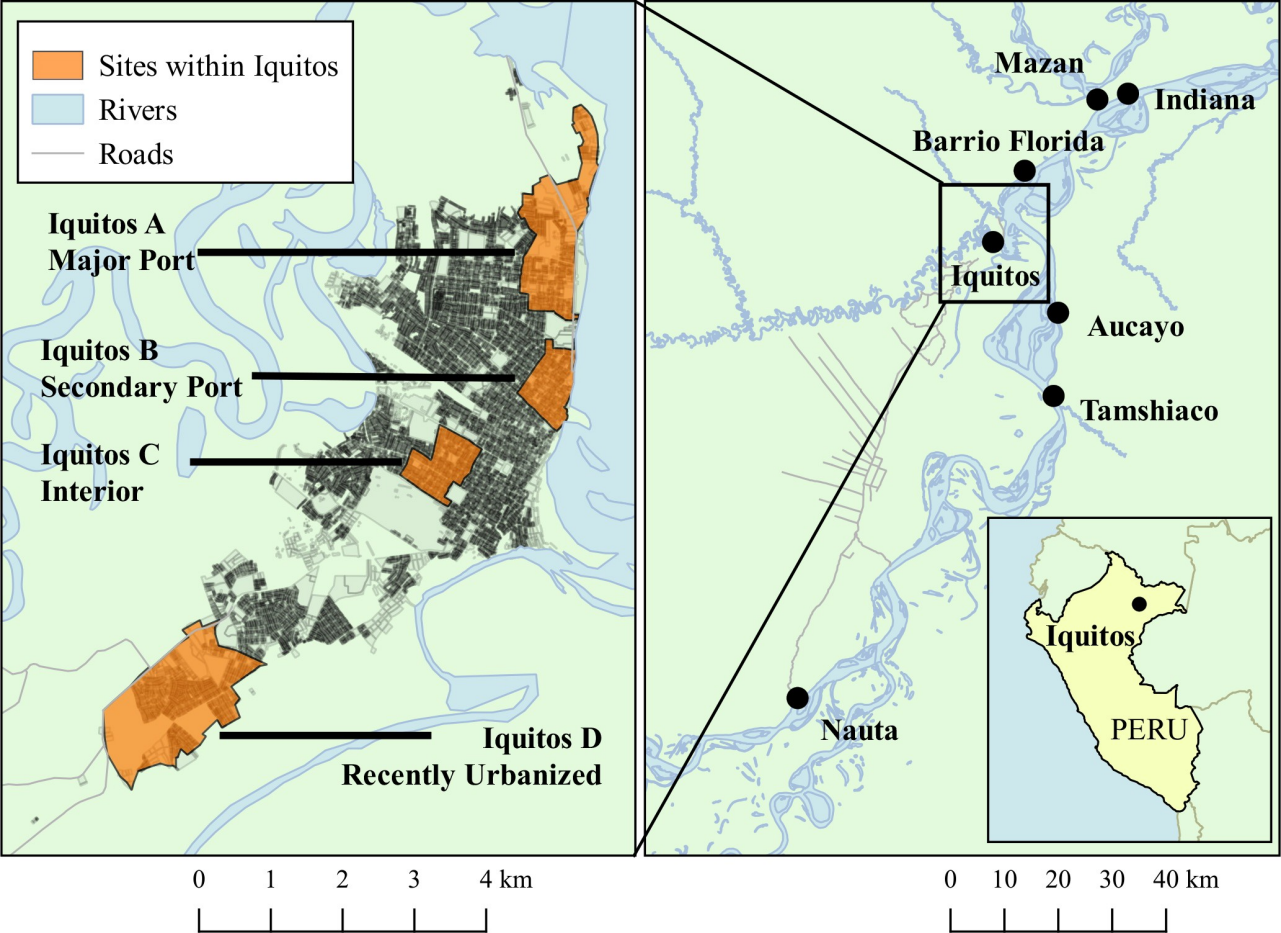
Map of *Ae*. *aegypti* collection sites in the Peruvian Amazon. We collected *Ae*. *aegypti* mosquitoes from seven population centers in the Peruvian Amazon: Iquitos, Nauta, Aucayo, Tamshiaco, Barrio Florida, Indiana, and Mazan. Collections from Indiana and Mazan were pooled into a single group to ensure > 20 individuals per site. In addition, we collected mosquitoes from four locations within Iquitos, two port sites and two interior non-port sites.

Mosquitoes were collected from the city of Iquitos, and the neighboring towns of Nauta (pop: 13,983), Indiana-Mazan (pop: 6,594), Barrio Florida (pop: 728), Tamshiaco (pop: 4,583), and Aucayo (pop: 806). **Fig 1**and **Table 1**summarize characteristics associated with each town. (The map in **Fig 1**was created in the program Quantum GIS [42] using shapefiles generated from prior research activities [43].) After asking permission to survey the household for *Ae*. *aegypti* mosquitoes, team members collected adult and immature mosquitoes either through aspiration of adults or larval surveys. Mosquito collection methods are described in detail elsewhere [44]. Within communities, we diversified mosquito sampling to the extent possible: we collected a maximum of 3 mosquitoes from any given household, and sampled 1 out of every 10 houses. More than 20 individuals were collected for each sampling location, although mosquitoes collected from Indiana (n = 18) and Mazan (n = 14) were grouped into one population to ensure adequate sample sizes for the calculation of inbreeding coefficient F_IS_, F_ST_, and Bayesian clustering analysis (described in detail below). Indiana and Mazan are only 1.2 km apart and our data indicated that mosquito populations in those two locations were genetically indistinguishable (F_ST_ = 0.032). The Euclidean distances between pairs of towns ranged from approximately 15km (Aucayo and Tamshiaco) to 125km (Nauta-Indiana/Mazan). (**S1 Table** demonstrates Euclidean pairwise distances between towns.)

**Table 1:** Characteristics of study sites.

City	Sub-Sample	N	Collection Date	Human population
Aucayo	-	20	Sept 2008	806
Barrio Florida	-	31	April 2008	728
Indiana-Mazan	-	32	Mar 2008	6,594
Nauta		55	May 2007, Mar 2008	13,983
Tamshiaco	-	76	Mar 2008	4,583
Iquitos	-	-	-	406,340
	Iquitos A—Port	21	May 2007, Mar 2008	-
	Iquitos B—Port	40	Mar 2008	-
	Iquitos C—Interior	35	May 2007, Feb, Mar 2008	-
	Iquitos D—Recently urbanized	29	Feb 2008	-

We profiled > 20 individuals per location, with multiple sampling sites within Iquitos. Mosquito collections took place in 2007 and 2008. Human population data was derived from the Peruvian National Census in 2007.

We collected mosquitoes from multiple locations in Iquitos to determine whether mosquitoes in port areas were more closely related to those in the surrounding towns (**Fig 1**). The location Iquitos A (Puerto Masusa) serves as a major hub of fluvial transit and predominantly harbors large barges, which carry cargo and passengers throughout the Peruvian Amazon distances up to ~500km. Iquitos B (Huequito) is a secondary fluvial port, primarily harboring medium-sized barges that also carry cargo and passengers, but that travel more locally (up to distances of ~250km) [24]. We also sampled mosquitoes from the interior of Iquitos (Iquitos C, district of Iquitos) and from a recently urbanized southern neighborhood near the start of the Iquitos-Nauta highway (Iquitos D, district of San Juan).

### DNA extraction and amplification

Methods used for genotyping mosquitoes with microsatellite markers are described by Wong et al [45]. Genomic DNA from the mosquito body was purified by potassium acetate/ethanol precipitation [46]. In a multiplex polymerase chain reaction (PCR), we amplified 8 previously described microsatellite markers [47, 48]. PCR products were diluted 1:60 or 1:40 in ddH_2_O, and submitted to the University of California Davis College of Agriculture and Environmental Sciences Genomics Facility for fragment analysis on an ABI 3730 XL capillary electrophoresis sequencer (Life Technology Corp.). GS600 LIZ size standard (Life Technology Corp.) was included with each sample to determine the size of individual peaks. ABI Peak Scanner software (Applera Corp., Norwalk, CT) was used to visualize resulting chromatograms. After identifying fragments, alleles were assigned using the *MsatAllele* package in R [49, 50].

### Microsatellite data analysis

We used the microsatellite data to calculate observed and expected heterozygosity values, in addition to inbreeding coefficients, F_IS_, for each site and genetic locus in Arlequin v3.5.2.2 [51]. We also tested for departure from Hardy-Weinberg equilibrium (exact test with 1,000,000 Markov chain steps and 100,000 dememorization steps) in Arlequin v3.5.2.2. A Sidak correction for multiple comparisons (for a total of 72 tests) was applied to determine significant deviation from Hardy-Weinberg equilibrium (p < 0.00071).

F_ST_ was calculated in Arlequin v3.5.2.2 (10, 000 permutations, significance level p < 0.05) [51]. We first conducted a pairwise F_ST_ analysis on all nine sampling locations to understand the degree of genetic differentiation among all sample locations including Iquitos. We then conducted a second pairwise F_ST_ analysis combining Iquitos samples to understand degree of connectivity between the city center and the five villages outside the city. Mantel tests conducted in the package *ade4* in R [50, 52] were used to test genetic isolation (10,000 permutations) by three different distance models for the data that combined Iquitos samples: 1) Euclidean distance, the shortest straight-line distance between two locations, 2) fluvial path distance, the river route between towns, and 3) shortest path distance, the shortest accessible fluvial or terrestrial route between two locations. Input F_ST_ values used to generate isolation by distance plots are shown in **S2 Table.**

We also developed a “Propagule Pressure Index,” combining the probability of *Ae*. *aegypti* infestation in different vehicle types with the frequency of travel between Iquitos and surrounding towns. We calculated infestation probabilities from data collected in 2013 across six different vehicle types common in Iquitos including: large and medium size barges, water taxis, speed boats, buses, and taxis [24]. Our results indicated that some vehicle types were consistently infested with *Ae*. *aegypti* across multiple months (71% of large barges, 35% of medium-sized barges, and 12.5% of buses). Simultaneous with entomological surveys, we interviewed vehicle drivers to determine the frequency of travel between Iquitos and surrounding towns for each vehicle type to estimate the approximate number of vehicles traveling to each town. The Propagule Pressure Index (PrPI) is calculated as follows:

PrPI *= Σ*_*j = 1*,*n*_ *(Σ*_*i = 1*,*n*_ *(γ*_*i*_*θ*_*i*_*))*

Where:

*γ*_*i*_ = probability of vehicle infestation

*θ*_*i*_ = number of trips from Iquitos to surrounding towns

*i* represents individual vehicles of a certain type

*j* represents different vehicle types (large barges, medium barges, and buses)

PrPI therefore represents the number of trips made between pairs of locations, weighted by the probability of *Ae*. *aegypti* infestation for each vehicle type. Values of PrPI were plotted against F_ST_ to visually explore the relationship between these two variables. However, no statistical tests were conducted due to low sample sizes—pairwise calculations of PrPI were only available between Iquitos and other towns, leaving a total of five observations.

We also estimated the genetic divergence of the populations by an analysis of molecular variance (AMOVA) in Alrequin v3.5.2.2 [51]. The geographic structure considered in this analysis is shown in **Table 1**; Aucayo, Barrio Florida, Indiana-Mazan, and Tamshiaco were counted as separated populations, but Iquitos sites were given hierarchical consideration. The total variation observed was attributed to differences between individuals within populations, and among populations. Accordingly, we calculated pairwise F statistic analogues characterizing the variation among individuals and populations (10,000 permutations, significance level p < 0.05).

We investigated the regional population structure of *Ae*. *aegypti* with the program Structure v. 2.3.4 [53]. Structure uses estimated allele frequencies to compute the likelihood that a given genotype originated from a genetic cluster. The result is that probabilistic estimates of population membership coefficients are assigned to each individual. Ancestral genetic admixture within an individual is observed when an individual has more than one population group is assigned. (For example, individual A is genetically admixed if the probability of belonging to group 1 is 0.6 and to group 2 is 0.4—the sum of these components is 0.6 + 0.4 = 1.)

In Structure, we used an admixture model with uncorrelated allele frequencies to avoid the risk of overestimating the number of populations (100,000 burn-ins and 200,000 Markov Chain Monte Carlo runs after the burn-in period). We started simulations with K = 10, to allow for the possibility of more genetic clusters than sampling locations (9 sampling locations including 4 Iquitos locations, and 5 towns), and then ran simulations for K values of 10 through 1. For each K, we ran 10 simulations to ensure consistency between runs, and used the log likelihood [53] and DeltaK method [54] to determine the most likely number of genetic clusters.

## Results

After applying a Sidak correction for multiple comparisons (p < 0.00071) for all populations, significant heterozygous deficits were detected in eight comparisons (both statistically significant and F_IS_ > 0.50) among four loci (B07, AC1, AG2, H08) (**Table 2**). These comparison were distributed across five sites including Barrio Florida (B07), Tamshiaco (B07), Iquitos A (AC1 and AG2), Iquitos C (B07 and H08), and Iquitos D (AC1 and B07). At a site-level, significant and low to moderate levels of heterozygous deficits were observed in Nauta, Tamshiaco, and all sites within Iquitos (F_IS_ range among these sites: 0.19629 to 0.37096).Of 72 tests, 45 (62.5%) loci from all populations were found to be in Hardy-Weinberg equilibrium. The majority of deviations were from Nauta and Tamshiaco; when those populations were excluded, 73.2% of loci (41 of 56) were in Hardy-Weinberg equilibrium.

**Table 2:** Summary of variation at 8 microsatellite loci by sampling location.

	A10	AC1	AC5	AG2	AG5	AT1	B07	H08	All loci
**Barrio Florida**									
N	26	25	25	26	29	27	26	26	
H_o_	0.46154	0.44000	0.48000	0.26923	0.51724	0.62963	0.07692*	0.11538*	
H_e_	0.52715	0.52980	0.68082	0.38612	0.59952	0.62753	0.33710	0.11237	
F_IS_	0.12664	0.17241	0.29927	0.30693	0.13934	0.00341	**0.77528**	0.0274	0.08561
**Aucayo**									
N	20	17	17	17	20	20	15	20	
H_o_	0.20000	0.52941	0.76471	0.11765	0.65000	0.50000	0.26667	0.30000	
H_e_	0.26154	0.66845	0.73262	0.29947	0.69103	0.66410	0.40460	0.43077	
F_IS_	0.24000	0.21311	0.04523	0.61446	0.06084	0.25197	0.34884	0.30909	0.14087
**Indiana-Mazan**									
N	32	30	30	30	32	32	30	31	
H_o_	0.31250	0.53333	0.63333	0.36667	0.65625	0.50000	0.26667	0.48387	
H_e_	0.33284	0.60791	0.69774	0.31921	0.68006	0.70685	0.39266	0.52089	
F_IS_	0.06203	0.12453	0.09375	0.15162	0.03556	0.29595	0.32460	0.07216	0.09449
**Nauta**									
N	55	32	55	55	55	55	51	55	
H_o_	0.32727*	0.40625*	0.80000*	0.54545*	0.65455	0.50909	0.23529*	0.32727	
H_e_	0.49274	0.70933	0.89091	0.64754	0.70859	0.72160	0.45913	0.34429	
F_IS_	0.33787	0.43119	0.10289	0.15888	0.07692	0.29642	0.49001	0.04985	0.19629^†^
**Tamshiaco**									
N	73	59	58	61	73	75	57	76	
H_o_	0.32877*	0.52542*	0.37931*	0.27869	0.54795*	0.54667*	0.05263*	0.28947*	
H_e_	0.55333	0.77329	0.72489	0.35564	0.80869	0.81002	0.46763	0.51098	
F_IS_	0.40751	0.3224	0.47891	0.21779	0.32394	0.3266	**0.88833**	0.43512	0.36043^†^
**Iquitos A**									
N	21	21	21	21	21	21	21	21	
H_o_	0.47619	0.19048*	0.42857*	0.33333*	0.80952	0.57143	0.28571	0.61905	
H_e_	0.47038	0.69570	0.84553	0.72125	0.82811	0.74448	0.47967	0.52846	
F_IS_	0.01266	**0.73109**	0.4993	**0.54397**	0.02299	0.23688	0.41032	0.17647	0.24141^†^
**Iquitos B**									
N	40	40	40	40	40	40	40	40	
H_o_	0.57500	0.62500	0.47500	0.50000	0.62500	0.72500	0.22500*	0.27500*	
H_e_	0.62120	0.70601	0.73228	0.48829	0.79715	0.80000	0.53165	0.56867	
F_IS_	0.01266	0.73109	0.49930	0.54397	0.02299	0.23688	0.41032	0.17647	0.25338^†^
**Iquitos C**									
N	35	35	35	35	35	35	35	35	
H_o_	0.51429	0.28571*	0.28571*	0.14286	0.62857	0.65714	0.28571*	0.31429*	
H_e_	0.59462	0.66832	0.67702	0.23561	0.80207	0.78012	0.77474	0.60828	
F_IS_	0.07526	0.11605	0.35425	0.02429	0.21812	0.09484	**0.57989**	**0.5196**	0.37096^†^
**Iquitos D**									
N	29	29	29	29	29	29	29	29	
H_o_	0.31034	0.34483*	0.55172*	0.31034*	0.65517	0.55172	0.24138*	0.37931	
H_e_	0.46038	0.71083	0.81307	0.53539	0.78826	0.78100	0.60436	0.49304	
F_IS_	0.32979	**0.51931**	0.3253	0.42466	0.17134	0.29725	**0.60484**	0.23383	0.33361^†^

N, sample size.

H_o_, observed heterozygosity.

H_e_, expected heterozygosity.

F_IS_, Inbreeding coefficient representing the reduction of heterozygosity in a subpopulation due to non-random mating.

Bolded F_IS_ values represent notable heterozygous deficits (F_IS_ > 0.50).

^†^ Statistically significant site-level F_IS_ after Sidak correction for multiple comparisons (p < 0.00071).

*Statistically significant deviation from Hardy-Weinberg equilibrium after Sidak correction for multiple comparisons (p < 0.00071).

Approximately 60% of loci were found to be in Hardy-Weinberg equilibrium (45 of 72 tests). Most of the significant deviations from Hardy-Weinberg equilibrium were from Nauta and Tamshiaco. Statistically significant (p < 0.00071) and notable heterozygous deficits (F_IS_ > 0.50) were observed in 8 in tests distributed across 5 sites.

Pairwise F_ST_ values demonstrated low to moderate differentiation for the majority of site pairs (**Table 3**). Mosquitoes from Barrio Florida and Nauta were significantly differentiated from all other sampling locations, with F_ST_ values ranging from 0.022 to 0.11. Barrio Florida mosquitoes had the highest F_ST_ values observed for all study sites (F_ST_ > 0.05 in 4 out of 8 pairwise comparisons). We also found significant genetic differentiation for 7 out of 8 pairwise comparisons for all other sites (Aucayo, Indiana/Mazan, Tamshiaco, Iquitos A, B, and C) except for Iquitos D. Iquitos samples generally showed a lower degree genetic differentiation both when comparing sites within Iquitos and sites outside of Iquitos. Notably, mosquitoes collected from Iquitos A and B (the major and secondary port for fluvial transit) had lower F_ST_ values than mosquitoes collected from the interior of Iquitos.

**Table 3:** Pairwise F_ST_ values between 9 sampling locations.

	Barrio Florida	Aucayo	Indiana/Mazan	Nauta	Tamshiaco	Iquitos A	Iquitos B	Iquitos C	Iquitos D
**Barrio Florida**	0								
**Aucayo**	0.0793*	0							
**Indiana/Mazan**	0.03882*	0.03062*	0						
**Nauta**	0.03857*	0.04908*	0.05776*	0					
**Tamshiaco**	0.05879*	0.04171*	0.05084*	0.02182*	0				
**Iquitos A**	0.09909*	0.04595*	0.05694*	0.06006*	0.03617*	0			
**Iquitos B**	0.03452*	0.0221*	0.01617*	0.04593*	0.01277*	0.03446*	0		
**Iquitos C**	0.11186*	0.05321*	0.08134*	0.07741*	0.02794*	0.05779*	0.0453*	0	
**Iquitos D**	0.03007*	0.01994	0.01682	0.02788*	0.00845	0.03074	0.00362	0.04614	0

* F_ST_ values statistically different from zero, indicating genetic differentiation (p < 0.05).

The fixation index, F_ST_, ranges from 0 to 1 and measures the degree of genetic relatedness between two pairs of populations by comparing the variation observed in the subpopulation with the variation observed in the total population. Values approaching 0 represent panmixia, whereas values approaching 1 represent complete genetic isolation (non-interbreeding populations). Our findings show low to moderate genetic differentiation, with the greatest degree of differentiation observed for Barrio Florida (highest F_ST_ values overall) and a lower degree of differentiation for Iquitos sites (lower F_ST_ values).

Genetic variation was compared within and between populations by AMOVA. Among all sites, approximately 71.5% of the variation was attributable to difference among individuals, whereas 24.9% of the variation was explained by individuals within populations, and just 4.1% among collections within Iquitos (**Table 4**).

**Fig 2:**
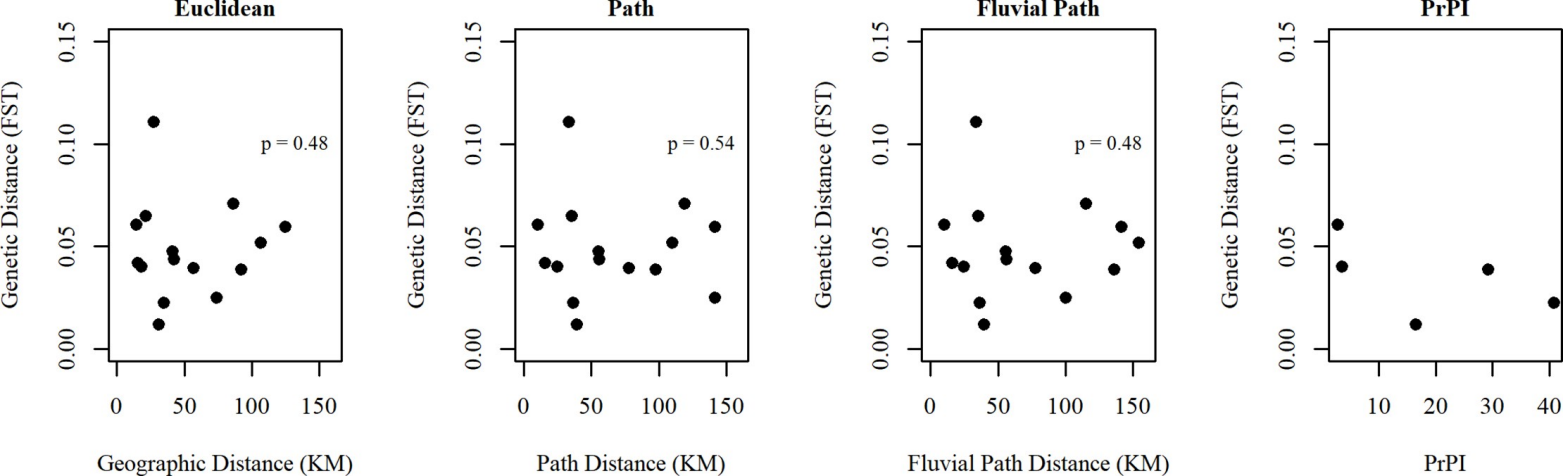
Isolation by distance models for three measures of geographic distance and for one measure of network distance for Iquitos and surrounding sites. A total of 15 pairwise comparisons are shown for Euclidean, Path, and Fluvial Path models. Only 5 pairwise comparisons were shown for the PrPI model, as transportation data were only available for traffic between Iquitos and surrounding towns (and not between other towns). P-values shown are Mantel probabilities.

Our analysis of isolation by distance showed no clear relationship between genetic distance (F_ST_) and geographic distance for the three standard distance models (**Fig 2**). We were unable to statistically evaluate the relationship between PrPI and genetic distances because of insufficient transportation network data.

**Table 4:** Analysis of Molecular Variance (AMOVA) of *Ae*. *aegypti* mosquitoes using 8 microsatellite loci.

Source of Variation	df	Sum of Squares	Variance components	Variation (%)	F-Statistic	P-value
Among groups	5	40.895	-0.00770	-0.38%	F_CT_ = 0.28534	0.61693
Among populations within groups	3	22.246	0.08117	4.05%	F_SC_ = 0.04035	<0.0001*
Among individuals within populations	330	801.514	0.49834	24.87%	F_IS_ = 0.25814	<0.0001*
Within individuals	339	485.500	1.43215	71.47%	F_IT_ = 0.28534	<0.0001*
**Total**	667	1350.155	2.00397			

F_CT_, differentiation among groups.

F_SC_, differentiation among populations within groups (Iquitos samples).

F_IS_, differentiation among individuals within populations.

F_IT_, differentiation within individuals.

*Statistically significant values (p < 0.05).

AMOVA results showed that 71.5% of the total variation observed was attributable to differences within individuals; only 4.1% of the total variation was attributable to differences among samples collected within Iquitos, indicating a lack of genetic structure within the city.

**Fig 3:**
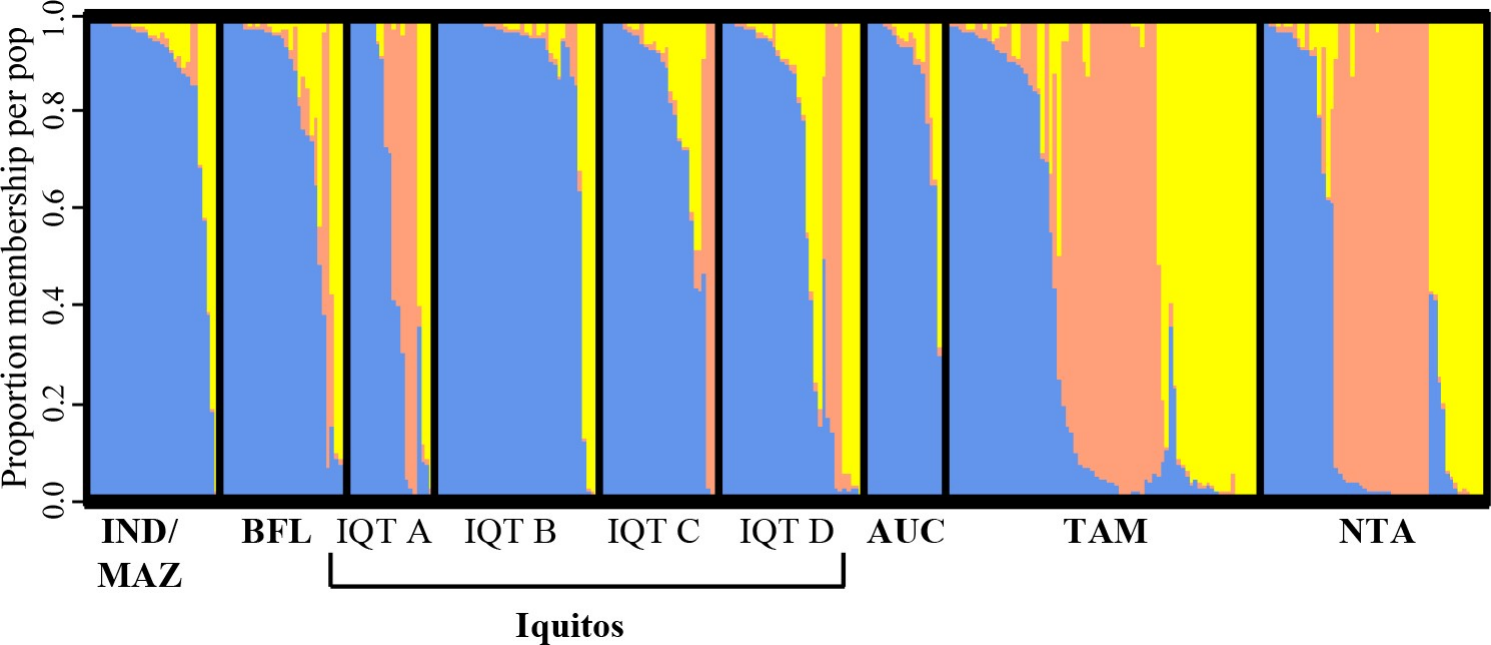
Structure diagram indicating the distribution of K = 3 genetic clusters across sampling locations. Individual mosquitoes are indicated by vertical bars and color denotes genetic group membership. The most likely number of populations were identified as being k = 3 different genetic groups. While the dominance of one genetic group (depicted in blue) in Iquitos B (IQTB), Indiana/Mazan (INDM), Aucayo (AUC), and Barrio Florida (BFL) indicates some genetic isolation at these sites, other sampling locations contain individuals from various genetic backgrounds.

Results from the DeltaK analysis of Structure output indicated that there were most likely three genetic clusters (**S1 Fig**). That is, the DeltaK statistic was highest for k = 3 groups (DeltaK = 9.5) in comparison with all other possible numbers of groups (DeltaK range: 0.18 to 1.6 for k = 1, k = 2, and k = 4 through 10). Structure results showed a clear pattern of genetic admixture from all the sample populations, suggesting steady gene flow within this region (**Fig 3**). Of 339 individual mosquitoes, 181 (53.4%) showed dominant haplotype membership associated with a single genetic group (represented in blue in **Fig 3**). Dominant haplotype membership (> 0.8) to the same group (depicted in blue in **Fig 3**) was observed among 84.4% of mosquitoes collected from Indiana/Mazan, 87.5% of mosquitos from Iquitos B, 75% of mosquitoes from Aucayo, and 64.5% of individuals from Barrio Florida. Mosquitoes collected from Tamshiaco and Nauta showed the greatest degree of ancestral admixture, with each of the three genetic groups represented approximately equally.

## Discussion

Our results support the hypothesis that *Ae*. *aegypti* long-distance dispersal is driven by human activity, with particular emphasis on boat traffic. Low F_ST_ values between Iquitos and surrounding towns in addition to our Structure results (i.e, the observation that no single population was exclusive to a single site) suggest mosquito mobility between human settlements. The pattern of mosquito gene flow also mirrors human transportation patterns: F_ST_ values for a regional port (Iquitos B) were overall lower than F_ST_ values collected from the interior of Iquitos (Iquitos C and D), implying more population mixing between Iquitos port mosquito populations and surrounding towns than between Iquitos interior populations and surrounding towns. Barrio Florida populations, in contrast, were the most genetically isolated compared to other towns. Iquitos is the major regional transportation hub, with frequent visits of barges and other types of boats from surrounding areas, whereas Barrio Florida is a small town (pop: ~800), receiving most of its traffic from vehicles unlikely to be infested with *Ae*. *aegypti* (e.g., speed boats and small water taxis) [24].

Some limitations should be noted when interpreting our data. It is possible that patterns of human traffic, and by extension, *Ae*. *aegypti* gene flow, have changed since mosquito samples were collected in 2008. More recent samples and better transportation data would be required to assess this possibility. Our limited data on human transport patterns also meant that we were only able to evaluate the Propagule Pressure Index by comparisons between Iquitos and surrounding towns. To that end, in some locations our sample sizes were low, particularly in Aucayo. Further, some scholars have argued that the allozyme methods to estimate population subdivision (e.g., F_ST_) are coarse measures of gene flow, and in some instances lead to erroneous conclusions [55]. Still, the results we present lay the groundwork for future population genetics approaches that may shed additional light on *Ae*. *aegypti* dispersal throughout the riverine landscape characteristic of this region. Fine-scale landscape genetics methods may be of particular interest to identify barriers to and pathways that facilitate dispersal [56].

Our previous studies in the Peruvian Amazon have shown that *Ae*. *aegypti* spreads farther along rivers than terrestrial routes [18], that *Ae*. *aegypti* spread is facilitated by certain vehicle types, large barges in particular (especially in comparison with terrestrial vehicles) [24], and that oviposition regularly occurs on boats while in transit [57]. When considered together with past findings, this exploration of *Ae*. *aegypti* population genetics provides additional evidence to support the hypothesis that boats are major drivers of the passive transport of *Ae*. *aegypti* mosquitoes over long distances (between cities > 5 km apart). In other regions, fluvial traffic has also been implicated as a driver of *Ae*. *aegypti* spread [58], but the present study is the first to illustrate the importance of boat traffic in the Peruvian Amazon specifically.

Consistent with other findings from Peru [59], our comparisons of *Ae*. *aegypti* genetic variation and geographic distance did not reveal a pattern of isolation by distance. At this spatial scale (sampling locations < 100km apart), network distance might be the best predictor of genetic mixing. Although both field ecology [16, 17] and population genetics studies [32, 60, 61] in the Americas have pointed to human transportation networks as a major driver of *Ae*. *aegypti* spread, to our knowledge, none of these studies linked ecological evidence with transportation and population genetics data (e.g., mosquito abundance collections, and data characterizing the number of vehicle trips between locations). The novel Propagule Pressure Index, PrPI, integrates these data by taking into account heterogeneity of *Ae*. *aegypti* infestation by vehicle type, in addition to the frequency of traffic between two locations. We were unable to statistically test for isolation by distance using PrPI because of limited sample sizes, however, the isolation by distance plot (**Fig 2**) is suggestive of a negative correlation between genetic and network distance. In other words, populations closer to one another in network space (as measured by PrPI) are likely to be more genetically related. Future studies with larger sample sizes are required to evaluate its utility.

Within Iquitos, *Ae*. *aegypti* genetic admixture of port mosquitoes and recently urbanized mosquitoes were similar, while interior mosquitoes had a different pattern of population membership. Within cities, mosquito population structure may be a function of availability of immature habitat, mosquito movement, or vector control programs that have differentially impacted various neighborhoods within the city. Perhaps, for example, insecticide application focused in the interior parts of the city act as barriers to population mixing, ultimately resulting in differing patterns of population group membership. This vector control explanation has been proposed in other settings [62].

Indeed, *Ae*. *aegypti* population structure in the Amazon has many other implications for dengue control programs. Our data suggest that *Ae*. *aegypti* are continually introduced to smaller surrounding communities from locations such as Iquitos that have stable and spatially diverse population structure. For example, the spread of insecticide resistance or vector competence genes would occur but could potentially lead to significant spatial heterogeneity. Significant spatial variation in vector competence has been reported previously [63], so understanding gene flow is critical for potential heterogeneity in transmission patterns. For new technologies that rely on population replacement (e.g., Wolbachia), it is possible that releases in large cities would be sufficient for spread to outlying areas.

An extension of our results is to ask, how does *Ae*. *aegypti* gene flow impact arboviral transmission dynamics? Dengue in the Peruvian Amazon has been characterized by transmission in large population centers with most cases observed from outlying communities assumed to be associated with frequent travel to these commercial hubs. The distribution of *Ae*. *aegypti* is driven by urbanization along highways, but infestation in communities along rivers is far less consistent spatially [18]. We hypothesize that establishment of *Ae*. *aegypti* in these communities is driven in part by repeated introductions as well as by local ecological characteristics such as water storage, management of containers, and microclimate. Over the last 20 years *Ae*. *aegypti* has spread to smaller communities, with significant implications for vector control programs which are already stretched to cover Iquitos and other larger cities. Dengue outbreaks are known to occur in several of the small riverine towns near Iquitos. Although these outbreaks are typically small and fleeting, the resources necessary for sustained control of *Ae*. *aegypti* (source reduction and larval control) are limited. Clearly, transmission in these smaller communities could lead to infections in travelers (and mosquitoes) that could then carry virus to other locations, thus serving as a mechanism of viral spread at broader scales.

## Supporting information

S1 Fig DeltaK analysis of Structure results. The DeltaK statistic captures the degree of change between the log probability of the data for consecutive values of K populations. Results showed the most likely number of groups to be K = 3 genetic clusters.
(TIFF)

S1 Table Pairwise distances between towns (Euclidean distance, fluvial path distance, shortest path distance, and PrPI). The number of visits between site pairs for large and medium barges were weighted according to the probability of their infestation (the columns Large Barges Weighted, Medium Barges Weighted). Our previous research demonstrated a 71% probability of infestation among large barges, 35% probability of infestation among medium barges, and 12.5% infestation among combis (taxis). PrPI is calculated by summing the number of visits by large and medium barges and combis, weighted for the probability of *Ae*. *aegypti* infestation.
(DOCX)

S2 Table F_ST_ table used to generate isolation by distance plots. Iquitos was counted as a single population because transportation data between different sites within Iquitos and surrounding towns was not available.
(DOCX)
