# Correction: The genetic structure of *Aedes aegypti* populations is driven by boat traffic in the Peruvian Amazon

**DOI:** 10.1371/journal.pntd.0010552

**Published:** 2022-06-15

**Authors:** The PLOS Neglected Tropical Diseases Staff

In Table 3, the following text should be removed from the legend:

Genetic variation was compared within and between populations by AMOVA. Among all sites, approximately 71.5% of the variation was attributable to differences among individuals, whereas 24.5% of the variation was explained by individuals within populations, and just 4.1% among collections within Iquitos (Table 4).

The above text should appear within the fourth paragraph of the Results section.

In the fourth paragraph of the Results section, the following sentence should be removed:

AMOVA results showed that 71.5% of the total variation observed was attributable to differences within individuals; only 4.1% of the total variation was attributable to differences among samples collected within Iquitos, indicating a lack of genetic structure within the city.

The above sentence should appear in the legend of Table 3. Please see Tables 3 and 4 and their correct legends here.

**Table 3:** Pairwise F_ST_ values between 9 sampling locations.

	Barrio Florida	Aucayo	Indiana/Mazan	Nauta	Tamshiaco	Iquitos A	Iquitos B	Iquitos C	Iquitos D
**Barrio Florida**	0								
**Aucayo**	0.0793*	0							
**Indiana/Mazan**	0.03882*	0.03062*	0						
**Nauta**	0.03857*	0.04908*	0.05776*	0					
**Tamshiaco**	0.05879*	0.04171*	0.05084*	0.02182*	0				
**Iquitos A**	0.09909*	0.04595*	0.05694*	0.06006*	0.03617*	0			
**Iquitos B**	0.03452*	0.0221*	0.01617*	0.04593*	0.01277*	0.03446*	0		
**Iquitos C**	0.11186*	0.05321*	0.08134*	0.07741*	0.02794*	0.05779*	0.0453*	0	
**Iquitos D**	0.03007*	0.01994	0.01682	0.02788*	0.00845	0.03074	0.00362	0.04614	0

* F_ST_ values statistically different from zero, indicating genetic differentiation (p *<* 0.05).

The fixation index, F_ST_, ranges from 0 to 1 and measures the degree of genetic relatedness between two pairs of populations by comparing the variation observed in the subpopulation with the variation observed in the total population. Values approaching 0 represent panmixia, whereas values approaching 1 represent complete genetic isolation (non-interbreeding populations). Our findings show low to moderate genetic differentiation, with the greatest degree of differentiation observed for Barrio Florida (highest F_ST_ values overall) and a lower degree of differentiation for Iquitos sites (lower F_ST_ values).

**Table 4:** Analysis of Molecular Variance (AMOVA) of *Ae*. *aegypti* mosquitoes using 8 microsatellite loci.

Source of Variation	df	Sum of Squares	Variance components	Variation (%)	F-Statistic	P-value
Among groups	5	40.895	-0.00770	-0.38%	F_CT_ = 0.28534	0.61693
Among populations within groups	3	22.246	0.08117	4.05%	F_SC_ = 0.04035	<0.0001*
Among individuals within populations	330	801.514	0.49834	24.87%	F_IS_ = 0.25814	<0.0001*
Within individuals	339	485.500	1.43215	71.47%	F_IT_ = 0.28534	<0.0001*
**Total**	667	1350.155	2.00397			

F_CT_, differentiation among groups.

F_SC_, differentiation among populations within groups (Iquitos samples).

F_IS_, differentiation among individuals within populations.

F_IT_, differentiation within individuals.

*Statistically significant values (p *<* 0.05).

AMOVA results showed that 71.5% of the total variation observed was attributable to differences within individuals; only 4.1% of the total variation was attributable to differences among samples collected within Iquitos, indicating a lack of genetic structure within the city.
