# Supplementary figures and images for: The genetic structure of Aedes aegypti populations is driven by boat traffic in the Peruvian Amazon
Source: PLoS Negl Trop Dis. 2019 Sep 18;13(9):e0007552. doi: 10.1371/journal.pntd.0007552 (PMC6750575; doi:10.1371/journal.pntd.0007552)

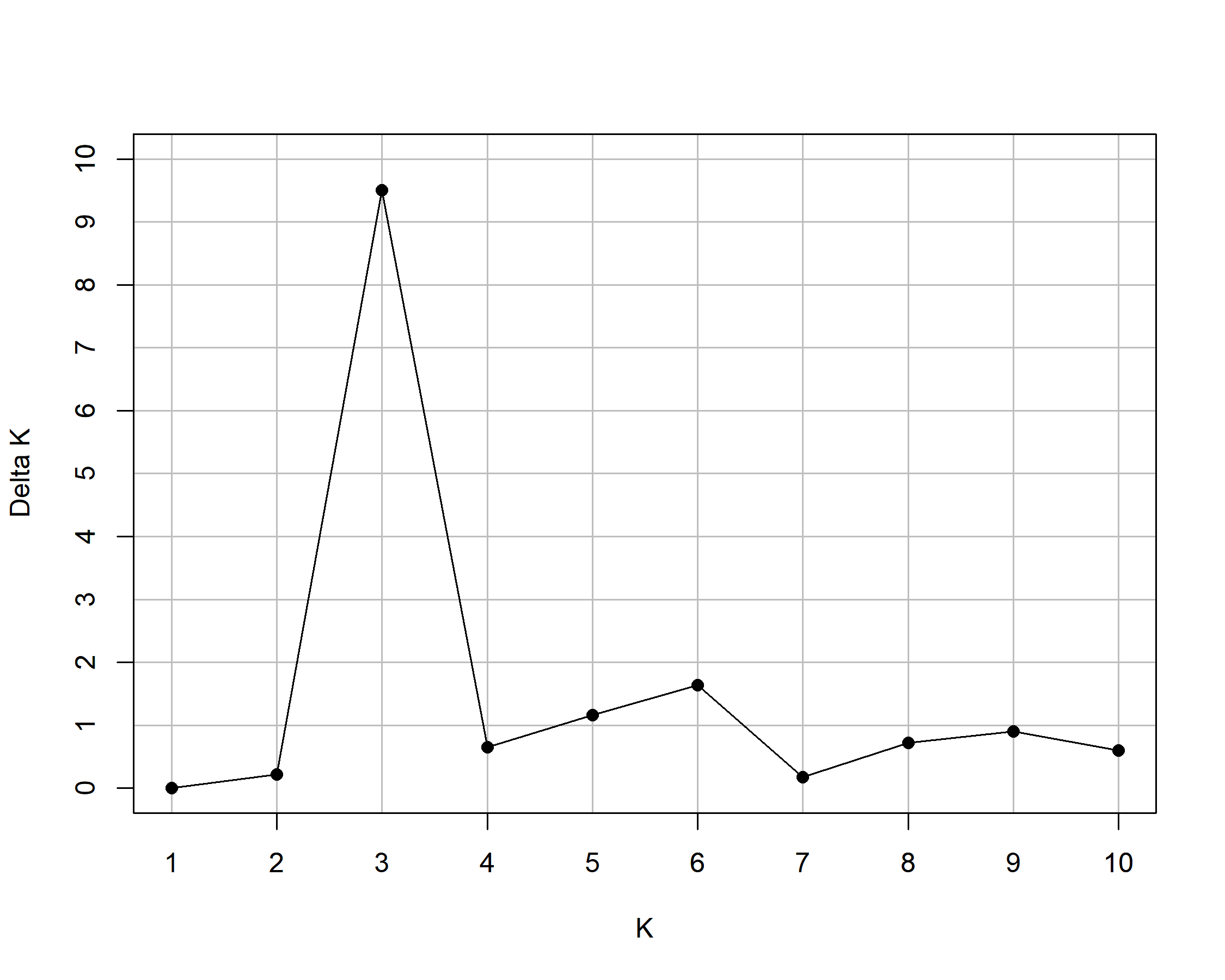

Supplement: S1 Fig — The DeltaK statistic captures the degree of change between the log probability of the data for consecutive values of K populations. Results showed the most likely number of groups to be K = 3 genetic clusters. (TIFF) [file pntd.0007552.s001.tiff]
