# Supplementary material for: The genetic structure of Aedes aegypti populations is driven by boat traffic in the Peruvian Amazon
Source: PLoS Negl Trop Dis. 2019 Sep 18;13(9):e0007552. doi: 10.1371/journal.pntd.0007552 (PMC6750575; doi:10.1371/journal.pntd.0007552)
Supplement: S1 Table — The number of visits between site pairs for large and medium barges were weighted according to the probability of their infestation (the columns Large Barges Weighted, Medium Barges Weighted). Our previous research demonstrated a 71% probability of infestation among large barges, 35% probability of infestation among medium barges, and 12.5% infestation among combis (taxis). PrPI is calculated by summing the number of visits by large and medium barges and combis, weighted for the probability of Ae. aegypti infestation. (DOCX) [file pntd.0007552.s002.docx]

| **Town Pair** | **Distance (km)** | **Fluvial Path Distance (km)** | **Shortest Path Distance (km)** | **No. Large Barges/ Month** | **No. Medium Barges/ Month** | **No. Combis/ Month** | **Large Barges Weighted** | **Medium Barges Weighted** | **Combis Weighted** | **PrPI** |
| --- | --- | --- | --- | --- | --- | --- | --- | --- | --- | --- |
| Iquitos-Barrio Florida | 14.354 | 9.978 | 9.978 | 0 | 8 | 0 | 0 | 2.8 | 0 | 2.8 |
| Iquitos-Aucayo | 17.812 | 24.436 | 24.436 | 3 | 4 | 0 | 2.1 | 1.4 | 0 | 3.5 |
| Iquitos-Tamshiaco | 30.65 | 39.252 | 39.252 | 3 | 41 | 0 | 2.1 | 14.4 | 0 | 16.5 |
| Iquitos-Indiana/Mazan | 34.74 | 36.321 | 36.321 | 6 | 104 | 0 | 4.3 | 36.4 | 0 | 40.7 |
| Iquitos-Nauta | 91.97 | 135.853 | 97.446 | 8 | 7 | 168 | 5.7 | 2.5 | 21 | 29.2 |
| Aucayo-Barrio Florida | 27.035 | 33.371 | 33.371 | - | - | - | - | - | - | - |
| Aucayo-Indiana/Mazan | 41.18 | 55.32 | 55.32 | - | - | - | - | - | - | - |
| Aucayo-Nauta | 86.246 | 115.023 | 118.795 | - | - | - | - | - | - | - |
| Aucayo-Tamshiaco | 15.439 | 15.834 | 15.834 | - | - | - | - | - | - | - |
| Barrio Florida- Indiana/Mazan | 21.1 | 35.24 | 35.24 | - | - | - | - | - | - | - |
| Barrio Florida-Nauta | 106.435 | 153.816 | 110.11 | - | - | - | - | - | - | - |
| Barrio Florida-Tamshiaco | 42.076 | 55.939 | 55.939 | - | - | - | - | - | - | - |
| Indiana/Mazan-Nauta | 124.8 | 141.183 | 141.248 | - | - | - | - | - | - | - |
| Indiana/Mazan-Tamshiaco | 56.51 | 77.65 | 77.65 | - | - | - | - | - | - | - |
| Nauta-Tamshiaco | 73.5 | 100.2 | 141.7 | - | - | - | - | - | - | - |

**S1 Table. Pairwise distances between towns (Euclidean distance, fluvial path distance, shortest path distance, and PrPI).**
