# Supplementary material for: The genetic structure of Aedes aegypti populations is driven by boat traffic in the Peruvian Amazon
Source: PLoS Negl Trop Dis. 2019 Sep 18;13(9):e0007552. doi: 10.1371/journal.pntd.0007552 (PMC6750575; doi:10.1371/journal.pntd.0007552)
Supplement: S2 Table — Iquitos was counted as a single population because transportation data between different sites within Iquitos and surrounding towns was not available. (DOCX) [file pntd.0007552.s003.docx]

**S2 Table. F_ST_ table used to generate isolation by distance plots.**

|  | **Barrio Florida** | **Aucayo** | **Indiana/Mazan** | **Nauta** | **Tamshiaco** | **Iquitos** |
| --- | --- | --- | --- | --- | --- | --- |
| **Barrio Florida** | 0 |  |  |  |  |  |
| **Aucayo** | 0.1107* | 0 |  |  |  |  |
| **Indiana/Mazan** | 0.06505* | 0.0476* | 0 |  |  |  |
| **Nauta** | 0.05182* | 0.07101* | 0.05953* | 0 |  |  |
| **Tamshiaco** | 0.04372* | 0.04202* | 0.03954* | 0.02525* | 0 |  |
| **Iquitos** | 0.06085* | 0.04019* | 0.02253* | 0.03902* | 0.01197* | 0 |

* F_ST_ values statistically different from zero, indicating genetic differentiation.
